# Cervicofacial Actinomycosis in the Pediatric Population: Presentation and Management

**DOI:** 10.1177/00034894211021273

**Published:** 2021-06-01

**Authors:** Karan Gandhi, Benjamin D. van der Woerd, M. Elise Graham, Michelle Barton, Julie E. Strychowsky

**Affiliations:** 1 Schulich School of Medicine and Dentistry, Western University, London, ON, Canada; 2 Department of Otolaryngology-Head and Neck Surgery, Western University, London, ON, Canada; 3 Department of Pediatrics, Western University, London, ON, Canada

**Keywords:** infection, anaerobic bacteria, children, intraoral excision, common childhood external ear problems

## Abstract

**Background:**

Infection caused by *Actinomyces* species is a rare cause of head and neck infection in children. This chronic cervicofacial infection can present with localized swelling, abscess formation, sinus drainage and can be complicated by osteomyelitis.

**Methods:**

Presented are 2 pediatric cases of secondary actinomycosis in the context of congenital lesions: 1 patient with a previously excised preauricular sinus and another with a persistent sublingual mass. A comprehensive literature search was conducted for reported cases of pediatric actinomycosis in the cervicofacial region.

**Results:**

Both cases presented were successfully treated with a combination of complete surgical excision of the lesions and prolonged antibiotic therapy. Thirty-four pediatric cases of cervicofacial actinomycosis are reviewed, 2 presented herein, and 32 from the published literature. There was equal gender distribution and the median age was 7.5 years. The most common site for infection was the submandibular area. Four (12%) of cases arose in pre-existing congenital lesions. Most patients were treated with penicillin-based antibiotics for a median duration of 6 months following surgical excision or debridement.

**Conclusions:**

Actinomycosis is a rare infection of the cervicofacial region; secondary infections arising from congenital lesions of the head and neck are even more rare. A previously excised pre-auricular sinus and a sublingual dermoid cyst are not previously reported sites of infection. Actinomycosis should be suspected in chronically draining sinuses of the head and neck region and confirmed through anaerobic culture. Osteomyelitis is a potential complication and magnetic resonance (MR) imaging is warranted. Long-term antibiotic therapy with a penicillin-based antibiotic and surgical excision should be considered.

## Introduction

Cervicofacial actinomycosis is a rare cause of head and neck abscesses in healthy children. It is a rare, slowly progressive, subacute, or chronic infection caused by the *Actinomyces* species of bacteria.^1^ *Actinomyces species* are gram positive filamentous anaerobic bacteria that can present with characteristic sulfur granules. These bacteria commonly colonize the human oral cavity, gastrointestinal, and genitourinary tract.^2^ Mucosal injury, poor oral hygiene, and immunosuppression have been implicated in pathogenesis of this disease. *Actinomyces* infection is commonly polymicrobial and requires prolonged anerobic culture for up to 15 days to grow.^3^ The most common species involved in human infection is *A. israeli* but there are at least 25 other species less commonly described in the literature identified by 16S ribosomal RNA sequencing.^4^

Infection is classically described as a chronic submandibular swelling but has been described in multiple regions in the head and neck.^5^ Actinomycosis can present with pain, fever, and weight loss and may be misdiagnosed as other granulomatous diseases or malignancy.^6^ Infections may be complicated by draining sinuses, osteomyelitis, and bacteremia.^5,7^ Its tendency to persist, spread to adjacent tissue, and cause significant morbidity if not treated for extended periods with a penicillin-based therapy makes it important to confirm the diagnosis and initiate appropriate management.^8^ Anerobic cultures are required for diagnosis and should be obtained in an atypical pediatric head or neck mass.^9^ Appropriate treatment often includes both medical management with penicillin-based antibiotics to treat a potentially polymicrobial infection, and surgery for extensive or recurrent disease.^10^ We present 2 cases of children with secondary cervicofacial actinomycosis in sites not previously described and review the literature on this rare infection in children. The first of these cases is the first report of *A. turicensis* cervicofacial infection in a child.

## Cases

Case 1: A previously healthy, immunized, 10-year-old female presented with a 1-year history of recurrently infected left pre-auricular sinus. She was referred to the pediatric otolaryngology service after previous surgical debridement. However, despite excision with dissection down to the helical cartilage, she subsequently developed a chronic infection post-operatively with poor response to short courses of multiple oral antibiotics including cephalexin, amoxicillin, and trimethoprim-sulfamethoxazole (TMP/SMX) for presumed cellulitis including possible MRSA. All wound swabs were negative, including MRSA screening. Anaerobic cultures had not been done.

**Figure 1.**
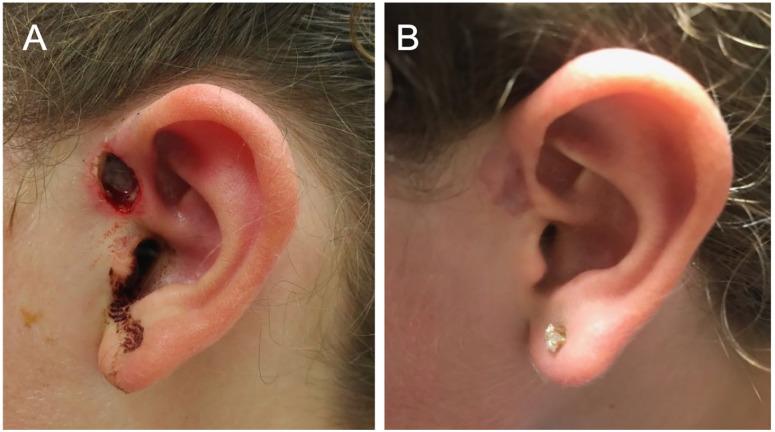
Post-operative image of preauricular sinus and healing of the sinus tract following therapy. (A) Recurrent preauricular sinus after wide local excision. Healing by secondary intention. (B) Healed pre-auricular sinus following a 6-month course of antibiotics post-operatively.

Physical examination at presentation revealed erythema and inflammation surrounding the previous surgical site. There was purulent drainage from this site which was sent for aerobic culture which showed gram positive cocci but no growth, likely due to TMP/SMX treatment at the time of the swab. Subsequently, the patient was consented for re-exploration and re-excision. In the operating room, a pre-auricular sinus was identified, which tracked down to the cartilaginous helix. A lacrimal probe was placed in the sinus to guide the dissection. Purulence was readily expressed, collected, and swabs were sent for both aerobic and anerobic culture. The sinus tract was circumferentially dissected out and resected en bloc at the root of the helix. The wound was copiously irrigated followed by a layered closure (Figure 1A). Pathology showed dermal granulation tissue with acute and chronic inflammation in keeping with secondary infection after the initial resection.

Gram-positive bacilli and gram-positive cocci were seen on microscopy, although only *Actinomyces turicensis* subsequently grew. Given polymicrobial findings on microscopy, the infectious diseases team recommended switching antibiotic coverage from amoxicillin to amoxicillin-clavulanate to cover both *Actinomyces spp.* and *Staphylococcus spp*. Subsequent magnetic resonance (MR) imaging confirmed that there was no evidence of osteomyelitis.

Surgical re-excision was completed 1 month later because of intermittent drainage from the operative site. The area was re-excised down to the cartilage. Soft tissue and perichondrium were scraped from the underlying cartilage. The area was packed and subsequently healed by secondary intention after 2 weeks. Gram stains of intraoperative specimens again identified gram positive bacilli and cocci and cultures subsequently grew only *A. turicensis*. The patient completed a 6-month course of amoxicillin-clavulanate post-operatively and has had no recurrence. When seen at the 6-month follow-up after completing antimicrobial therapy, there was no contour defect or evidence of ongoing infection (Figure 1B).

**Figure 2.**
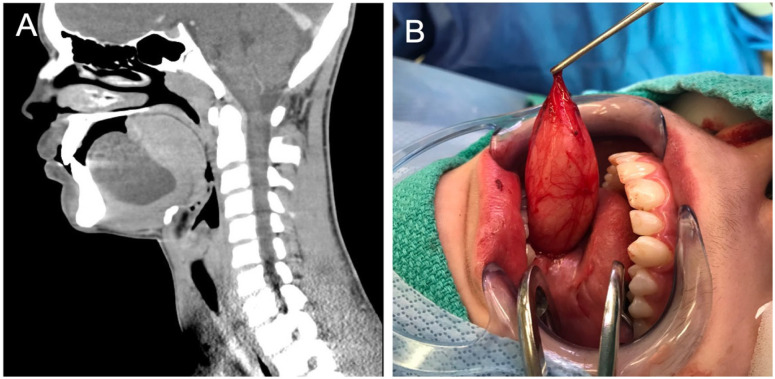
CT image and intraoperative image of the sublingual mass. (A) Sagittal CT view showing a ~5 cm × 5 cm sublingual mass. (B) Case 2: Intra-operative image of sublingual mass excision. Dermoid cyst measured ~5 cm × 5 cm.

Case 2: A 14-year-old, immunized, male presented with a history of a sublingual mass initially thought to represent a plunging ranula. Contrast enhanced CT neck showed a 5 cm cystic mass displacing the tongue superiorly (Figure 2A). The mass was first managed with decompression and marsupialization under local anesthetic. Aspirates sent for aerobic culture were negative. No anerobic cultures were requested. Clindamycin and chlorhexidine mouthwash were given for antimicrobial prophylaxis following the procedure. There were no signs of recurrence at the 1-week follow-up.

The patient presented to the emergency department 2 months later with concerns the lesion was increasing in size following trauma to the face and jaw. Muffling of the voice was noted. The mass was decompressed and 80 mL of yellow cloudy fluid was collected and sent for cytology, culture, and sensitivity. A 7-day course of amoxicillin was prescribed empirically for oral infection secondary to a congenital mass. There was no recurrence at the 3-week follow-up. At 5 months there was recurrence of sublingual swelling and the area was again aspirated and sent for both aerobic and anerobic cultures. This aspirate grew only *Actinomyces spp.* which was treated with 6-months of amoxicillin therapy. MRI confirmed that there was no bony involvement suggesting osteomyelitis. Another aspiration was needed 4 months later, this grew *Veillonella spp*. in addition to *Actinomyces spp*. Antibiotic coverage was broadened to high-dose amoxicillin-clavulanate. Due to the persistent infection and recurrence of swelling surgical excision was done. The pathology-confirmed dermoid cyst (Figure 2B) was completely excised and high-dose amoxicillin-clavulanate was extended for 3 months post-operatively. Total duration of antibiotic therapy was 9 months. There were no signs of recurrence 2 months post-operatively.

## Methods

A search of the published literature up to March 28, 2020 was conducted using the online search databases PubMed, EMBASE, and Medline. The search used MeSH terms or free text phrases that combined infection-specific terms (*Actinomyces* AND actinomycosis) with condition-specific terms (head and neck OR head OR skull, neck OR neck muscles; OR cervicofacial OR facial OR face OR cervical; OR mandibular, sublingual OR oral) and age-specific terms (child, neonate, pediatric, pediatric, boy, girl). Cochrane and Web of Science were also searched but no relevant, non-duplicate articles were identified. Articles selected for review were written in English and included confirmed cases of actinomycosis restricted to the head and neck in patients under 18 years of age. Abstracts and full articles were reviewed by 2 authors independently (KG and BVW). References of included articles were reviewed to identify additional reports that were not included in the original search.

Articles were excluded if they were not specific to the management of pediatric head and neck actinomycosis, if they reported on immunocompromised hosts, or if they specifically addressed actinomycosis of the tonsils, thyroid, intracranial, or dental actinomycosis as these have a different presentation than cervicofacial actinomyces infection. Demographic, clinical, microbiological, management, and outcome data were extracted using a standardized spreadsheet document.

## Results

**Figure 3.**
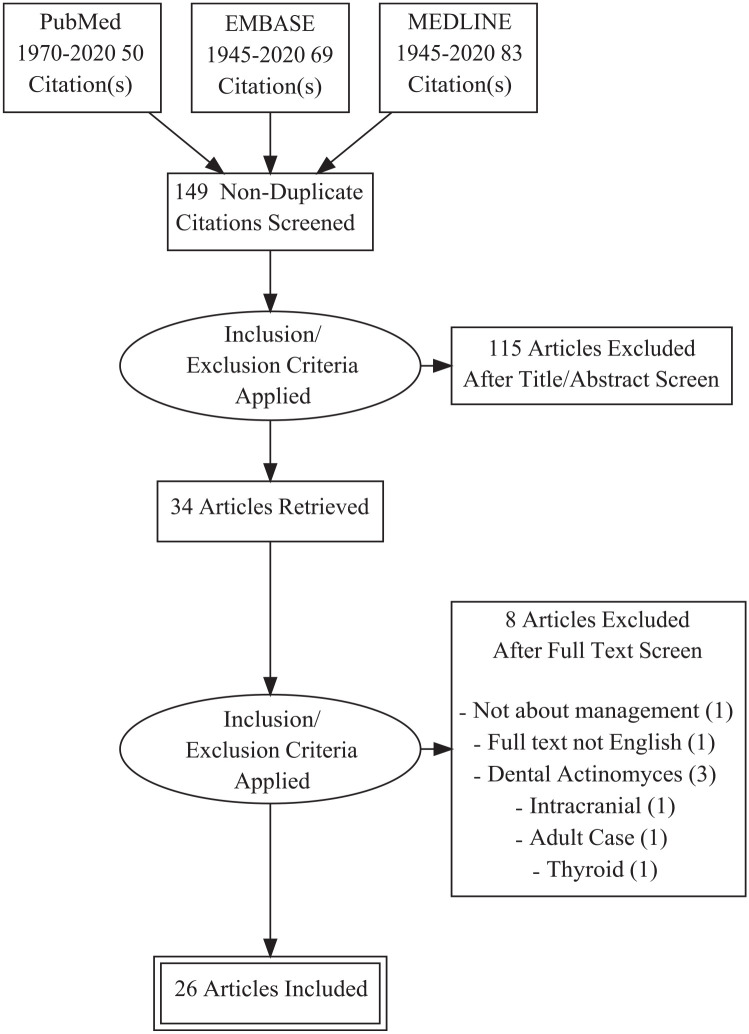
Flow diagram showing the literature review process for this literature review. The diagram shows the process by which studies were incorporated into the results of the literature search.

With our pre-defined search criteria, we identified a total of 149 English language articles. The authors excluded 115 articles during title and abstract screening; 34 articles received full text review and 26 were included in the final review (Figure 3). The 8 articles that did not fulfill study criteria after receiving full text review were excluded for the following reasons: site not qualifying (N = 6), non-English language (N = 1), and management not reported (N = 1).

From the 26 articles, 32 patient cases were reported, 16 (50%) male, 15 (48%) female, and 1 article (2%) where gender was not specified. The median age was 7.5 years, with a range of 20 months to 17 years. The most common site of infection was the mandibular region accounting for 15 cases (48%), 8 patients (25%) had infections in the cervical region, and 2 patients (6%) had an infra-auricular infection. Other sites of infection included the labial mucosa, temporal/temporo-facial, mastoid, parotid, temporal bone, and middle ear, which accounted for 7 cases (22%) combined. Literature review showed 2 (6%) cases of actinomycosis in the setting of congenital lesions, first in the setting of a dermoid cyst and the other in a pyriform sinus fistula. Along with our cases, actinomycosis arising in congenital head and neck lesions accounted for 4 (12%) of head and neck infections. Actinomycosis most commonly presented with a cervicofacial mass in 29 cases (91%). Of the 23 cases that included information about patient symptoms, 15 (65%) had pain at the site. Sinus tracts developed in 6 cases (19%) and 13 cases (41%) reported features consistent with osteomyelitis. Otalgia or otorrhea were reported in 3 cases, but only presented in middle ear infections. The most common organisms were *A. israelii*; reported in 8 cases (28%) and *A. odontolyticus*; reported in 4 cases (13%). *A. neuii, A. naeslundii* and were reported in 1 case each. The organism was unspecified in 18 cases (56%).

Penicillin-based therapy was given in 30 cases (94%), this included penicillin G/V, amoxicillin, and ampicillin. Two children (6%) received no penicillin-based antibiotics during treatment. Antibiotics used in these cases were clindamycin or cefuroxime and metronidazole. Seven of the 30 children (23%) who received penicillin-based therapy also received non-penicillin-based antibiotics for some or most of their antimicrobial therapy. Non-penicillin antibiotics in these cases included: meropenem, clindamycin, doxycycline, pristinamycine cefazolin, clarithromycin, and azithromycin. Surgery or aspiration was performed in 24 cases (75%) and no procedures were performed in 8 cases (25%). Antibiotic therapy was continued for a median of 6 months with a range of 1 week to 25 months, with some patients receiving antibiotic therapy intermittently over years due to recurrence. All included case reports reported either cure of the infection or loss to follow-up. This data is summarized in Table 1.

**Table 1.** Data from 34 Cases of Pediatric Cervicofacial Actinomycosis.

Paper	Age/gender	Site of infection	Presentation	*Actinomyces species* (spp)	Antibiotic (duration)	Surgery	Primary or secondary infection
Present study Case 1, 2019	10/F	Pre-auricular sinus	Inflammation Sinus tract	*Actinomyces turicensis*	Amoxicillin/clavulanate (6 mo)	Excision	Secondary to congenital lesion
Present study Case 2, 2019	15/M	Sublingual	Painless mass	Not stated (NS)	Amoxicillin/clavulanate (9 mo)	Decompression Marsupialization	Secondary to congenital lesion
Drake and Holt, 1970^24^	3/F	Cervical	Mass	*A. israelii*	Cloxacillin (0.25 mo) Penicillin (3.5 mo)	Drainage	Primary
	5/F	Cervical	Mass	*A. israelii*	Penicillin (3.3 mo)	Aspiration Resection	Primary
Roveda, 1973^25^	17/M	Parotid	Painful mass	NS	Penicillin (0.5 mo)	Drainage	Primary
Walker et al, 1981^26^	7/F	Mandibular	Mass Fever Osteomyelitis	*A. israelii*	Penicillin (3 mo)	Curette	Primary
Badgett and Adams, 1987^27^	5/F	Submandibular	Painful mass Osteomyelitis	NS	Clindamycin (10 mo)	Drainage	Primary
Feder, 1990^28^	6/M	Mandibular	Painful mass Fever Osteomyelitis	*A. israelii*	Penicillin (3.5 mo)	Drainage	Primary
	13/M	Mandibular	Painful mass	*A. israelii*	Penicillin (4 mo)	Drainage	Primary
Carrau et al, 1993^29^	6/M	Temporofacial	Painless mass	NS	Penicillin (9 mo)	Excision	Primary
Foster et al, 1993^30^	11/M	Submental	Mass	NS	Penicillin (7 mo)	Curette	Primary
Altundal et al, 2000^31^	10/M	Submandibular	Painful mass Sinus tract Osteomyelitis	NS	Penicillin (19.25 mo)	—	Primary
Schwartz and Wilson, 2001^32^	17/M	Mandibular	Painful mass	NS	Amoxicillin (6 mo)	Surgical Incision	Primary
Hong et al, 2004^33^	9/M	Posterior neck	Mass	NS	Penicillin (0.25 mo)	—	Primary
Sobol et al, 2004^34^	12/F	Temporal bone	Otalgia, otorrhea Hearing loss Osteomyelitis	NS	Penicillin (6 months)	Excision	Primary
Robinson et al^22^	7/F	Mandibular	Painful mass Osteomyelitis	NS (2 species)	Penicillin (7.5 mo) Clindamycin (0.5 mo) Doxycycline (3 mo) Clindamycin (8 mo)	Debridement	Primary
	4/F	Mandibular	Mass Osteomyelitis	*A. israelii*	Penicillin, Clindamycin, Clarithromycin, and Metronidazole Azithromycin (~4 y)*	Debridement	Primary
	3/M	Mandibular	Painful mass Osteomyelitis Sinus tract	*A. naeslundii*	Cefazolin Penicillin Clindamycin Amoxicillin/clavulanate Metronidazole (22 mo)*	Biopsies Debridement Sequestrectomy	Primary
	4/F	Mandibular	Painful mass Osteomyelitis	*A. israelii*	Amoxicillin (6 mo) Clindamycin (7 mo) Amoxicillin/clavulanate (12 mo)	Sequestrectomy Mandibulectomy Debridement	Primary
Mehta et al, 2007^35^	11/M	Temporal bone	Otorrhea, otalgia Mass Osteomyelitis	NS	Ampicillin/Sulbactam (2.25 mo)	Labyrinthectomy Debridement	Primary
Ciftologan et al, 2009^36^	9/M	Submandibular	Painless mass	NS	Amoxicillin (3 mo)	Excision	Primary
Gazzano et al, 2010^37^	8**	Middle ear	Otorrhea	NS	Amoxicillin (5.75M) Amoxicillin + Pristinamycine (2 mo)	Excision	Primary
Hung et al, 2014^38^	7/F	Posterior cervical region	Painful mass Neck pain	NS	Penicillin (12 mo)	Partial excision	Primary
Lezcano et al, 2014^39^	10/M	Mastoid	Painful Mass Fever	NS	Meropenem (1.5 mo) Amoxicillin/clavulanate (6 mo)	Mastoidectomy	Primary
Thacker and Mary Healy, 2014^40^	16/F	Submandibular	Painless mass	NS	Penicillin (7 mo)	—	Primary
Verma et al, 2014^41^	6/F	Labial mucosa	Mass Sinus tracts	*A. israelii*	Amoxicillin/clavulanate (1 mo)	—	Secondary to congenital lesion
Walther et al, 2014^42^	1.67/F	Cervical region	Painless mass Sinus tract	*A. neuii*	Amoxicillin/clavulanate (6 mo)	Excision	Primary
Chatterjee et al, 2015^43^	13/M	Infra-auricular	Painful mass Facial swelling Osteomyelitis	NS	Cefuroxime/metronidazole	Incisional Biopsy Resection	Primary
Prajapati et al, 2016^44^	8/M	Cervical spine	Painful ulcer Osteomyelitis	NS	Ampicillin/sulbactam Penicillin (1 mo)	—	Primary
Sama et al^8^	11/F	Infra-auricular	Painless mass Sinus tracts	NS Others	Penicillin	Debulking drainage	Primary
Yanagisawa et al, 2017^45^	2/M	Cervical	Painless Mass Sinus tract	*A. odontolyticus*	Ampicillin/sulbactam (0.5M) Amoxicillin/clavulanate (6 mo)	Biopsy	Secondary to congenital lesion
Glass et al, 2019^46^	5/M	Submandibular	Painless Mass Odontalgia Osteomyelitis	*A. odontolyticus*	Amoxicillin/clavulanate (7.5 mo)	Debridement	Primary
	5/F	Submandibular	Odontalgia Mass Osteomyelitis	*A. odontolyticus*	Amoxicillin/clavulanate (6 mo)	Drainage	Primary
Savoca et al, 2019^47^	10/F	Cervical	Painful mass	*A. odontolyticus*	Clindamycin* Amoxicillin (2 mo) Penicillin (7.5 mo)	Biopsy	Primary

Abbreviations: F, female; M, male.

* Not included in average duration of treatment calculation as the exact duration of treatment was not reported.

** Gender not specified.

## Discussion

We present 2 cases of pediatric cervicofacial actinomycosis in the setting of congenital lesions representing the first report of actinomycosis at each respective site, although not the first report of infections arising from congenital lesions. *Actinomyces* species are a part of the normal oral flora and infection typically ensues when the host’s mucosal barriers or immune responses are compromised, which is likely the source of the sublingual infection reported. We postulate that the pre-auricular sinus became infected from the drooling of oral secretions during sleep. It is possible that in the case of the sublingual cyst actinomycosis could have developed spontaneously from previous aspiration or a microscopic breach in its mucosal surface that may have happened with the rapid growth of the cyst and possible thinning of its wall. Alternatively, the cyst could have become secondarily infected at the time of the initial surgery. Both children had polymicrobial infection, which is commonly present in actinomycosis and can make diagnosis more challanging.^11^

Actinomycosis is rare in childhood but more common in middle aged individuals. It can mimic a wide variety of diseases, including malignancy and tuberculosis and presents with an array of symptoms making diagnosis challenging.^12^ Cervicofacial presentations include localized swelling often in the mandibular area with or without abscess formation, fibrosis, and sinus drainage.^3,13^ *A. israelli* is the most common species known to cause infection in humans and was identified as the leading species causing pediatric cervicofacial actinomycosis in this review. Treatment is often comprised of surgical debridement followed by long-term penicillin-based therapy.

Pre-auricular sinuses are common congenital abnormalities that are thought to be the consequence of incomplete fusion of the 6 auditory Hillocks of His.^14^ These indentations near the anterior margin of the helix may become secondarily infected, most commonly with *Staphylococcus aureus.*^15^ As *Actinomyces* also causes sinus tract formation, it is possible that the infection may have created a de novo tract following successful and complete excision of the original preauricular sinus tract. Despite its rarity, *Actinomyces spp* should be considered in chronically draining sinuses, which show poor response to short courses of anti-Staphylococcal antibiotic therapy. Early diagnosis and treatment are important as long-term antibiotic therapy is important and serious complications, such as osteomyelitis, may ensue. In such cases, sending purulent secretions, rather than swabs, for cultures, especially anerobic cultures, maximizes detection of the organism. This allows for targeted antimicrobial therapy with a better chance of clearing the infection. Complete surgical excision of the pre-auricular pit along with the sinus tract is important to definitively manage recurrently infected sinuses.^15^ Wounds are closed primarily in most cases of surgical excision of pre-auricular sinuses. However, for actinomycoses with draining sinuses, the wounds should be packed for optimal healing by secondary intention.^16^

Dermoid cysts are benign lesions that can occur in the head and neck region.^17^ They are midline lesions resulting from sequestration of ectodermal tissue along embryonic fusion planes.^18^ This is the first report of a sublingual dermoid cyst infected with *Actinomyces spp*. As the earliest sampling taken from this cyst was not sent under anerobic conditions, the failure to grow actinomyces from that sample does not exclude its presence. We cannot determine if this lesion was spontaneously infected. The lesion was decompressed and marsupialized in clinic but failed to resolve and subsequently needed multiple aspirations. The failure of this cyst to resolve following initial surgical management and need for multiple aspirations raised concerns for secondary infection. Given the location of the dermoid cyst, there were also concerns for difficulties with speech, swallowing, mastication, and airway obstruction.^19,20^ The growth of *Actinomyces spp* directed targeted amoxicillin therapy. Despite antibiotic treatment and multiple aspirations, the lesion continued to enlarge. As a result, complete surgical excision of the cyst was necessary (Figure 2). An additional 3 months of antibiotic therapy was prescribed post-operatively, after which time there was complete resolution of the infection.

Cervicofacial actinomycoses commonly presents with a painful mass or swelling, with up to 50% arising in the mandibular region.^3^ However, the absence of pain should not be used to exclude the diagnosis, as up to one-third of cases may not present with pain. Osteomyelitis rarely complicates head and neck infections caused by *S.aureus* and other common acute pyogenic organisms. In contrast, osteomyelitis is a recognized complication of cervicofacial actinomycosis occurring in 38% of pediatric cases reported in the literature ; 67% of these bony infections involved the mandible.^21^ Infections complicated by bony infection may be challenging to clear and require extended antimicrobial therapy. Given the frequency of this complication and clinically silent pattern among reported cases, MRI should be done to exclude bony involvement. Although sinus tract formation is an important characteristic feature, we found that it only occurred in 16% pediatric reports and emphasizes that most pediatric cases do not demonstrate this distinguishing feature. It therefore emphasizes the importance of cultures in maximizing detection if pathognomonic features are not present. Actinomyces can form abscesses and tracts, which made it challenging in our first case to determine whether the tract was from previous incomplete excision of a congenital preauricular sinus tract or novel from the unsuspected actinomyces, however, symptoms only resolved after complete surgical excision and prolonged antibiotics.^3^

Treatment with a penicillin-based regimen for up to 6 months is recommended therapy in uncomplicated disease. Treatment may need to be longer if complications, including osteomyelitis, occur. Most cases of actinomycosis in the literature were treated with a penicillin-based therapy, including penicillin G, penicillin V, and amoxicillin. Co-infection with other organisms from skin such as *S. aureus* as well as oral flora may occur. In such cases, the cultures should guide the need for combination therapy. Both of our cases required beta-lactamase inhibitor combinations to address coinfections.

Surgical debridement, in conjunction with prolonged antibiotic therapy (6-12 months), was the most common management approach used in the literature and is recommended for congenital lesions that have become secondarily infected. Therapy for osteomyelitis was typically longer in duration than for uncomplicated infection.^22^ Tetracycline, erythromycin, clindamycin, or third generation cephalosporins can also be used, especially in cases where the patient has a penicillin allergy.^23^

## Conclusions

Cervicofacial actinomyces is a rare but important infection in children and most often arises in the mandibular region. Anaerobic cultures of exudate facilitate early diagnosis. Given this organism’s propensity to spread to bone and to form sinuses, MR imaging is recommended to ensure that there is no bony involvement. Microbiologic confirmation of this infection allows long-term, targeted, penicillin-based therapy for extended periods up to 6 months or longer if complications occur. These chronic infections are best managed with a combination of penicillin-based antibiotics and surgery.
